# Genome Sequence of *Geothermobacter* sp. Strain HR-1, an Iron Reducer from the Lō‘ihi Seamount, Hawai’i

**DOI:** 10.1128/genomeA.00339-18

**Published:** 2018-05-24

**Authors:** Hillary Smith, Karla Abuyen, Jason Tremblay, Pratixaben Savalia, Ileana Pérez-Rodríguez, David Emerson, Benjamin Tully, Jan Amend

**Affiliations:** a Community College Cultivation Cohort, University of Southern California, Los Angeles, California, USA; b Department of Earth Sciences, University of Southern California, Los Angeles, California, USA; c Center for Dark Energy Biosphere Investigations, University of Southern California, Los Angeles, California, USA; d Department of Earth and Environmental Science, University of Pennsylvania, Philadelphia, Pennsylvania, USA; e Bigelow Laboratory for Ocean Sciences, East Boothbay, Maine, USA; f Department of Biological Sciences, University of Southern California, Los Angeles, California, USA

## Abstract

Geothermobacter sp. strain HR-1 was isolated from the Lō‘ihi Seamount vent system in the Pacific Ocean at a depth of 1,000 m. Reported here is its 3.84-Mb genome sequence.

## GENOME ANNOUNCEMENT

Lō‘ihi Seamount is an active hydrothermal vent system rich in diffuse fluids containing Fe(II) (1). Extensive microbial mats of lithoautotrophic Fe-oxidizing bacteria dominated by *Zetaproteobacteria* proliferate around these vents and precipitate large amounts of poorly crystalline, ferrihydrite-like Fe-oxyhydroxide (2). Iron-reducing microorganisms are able to respire those Fe(III) precipitates, thus playing an important role in the biogeochemical cycling of iron. Here, we present the genome sequence of Geothermobacter sp. strain HR-1, a strain isolated from the Lō‘ihi Seamount hydrothermal vent system. The genus Geothermobacter is related to other iron reducers isolated from geographically distant locations in the Pacific Ocean (3, 4), so investigations into HR-1 may offer insight on the role this genus plays in seafloor iron cycling.

DNA extraction from strain HR-1 was performed using the UltraClean microbial DNA isolation kit (Mo Bio, Inc., Carlsbad, CA), following the manufacturer’s protocol, and sequenced using an Illumina MiSeq 2 × 300-bp platform at Molecular Research (Shallowater, TX, USA). A total of 5,278,368 raw paired-end reads were generated with an average DNA fragment length of 815 bp. Trimmomatic version 0.33 (5) was used to remove primers, the first 10 bp, and regions with low quality scores (Q < 28). The resulting 4,594,900 high-quality paired-end sequences were assembled using SPAdes version 3.10.1 (6), creating 386 contigs. The 73 longest contigs were selected for inclusion in the genome based on a delineation in the contig coverage value (∼190× coverage). The Geothermobacter sp. HR-1 genome is 3.84 Mb in length and has an *N_50_* value of 74,356 bp and a GC content of 59.21%. Annotation was performed using the NCBI Prokaryotic Genome Annotation Pipeline version 4.1, resulting in 3,325 coding DNA sequences (CDSs), 6 rRNAs (2 copies each of the 5S, 16S, and 23S genes), and 50 tRNAs. Based on CheckM version 1.0.3 (7), the genome is estimated to be 98.71% complete due to the presence of 245 of the 247 markers from the Deltaproteobacteria lineage set.

Genomic analysis indicates that strain HR-1 has metabolic potential similar to that of its closest relative (99.6% 16S rRNA sequence identity), Geothermobacter sp. strain EPR-M. Both genomes include genes coding for hydrogen oxidation (*hyaABC*) and the fermentation of acetate and formate (4). Although as yet unconfirmed during culture experiments, the likelihood that strain HR-1 is prototrophic for several essential metabolic cofactors and capable of motility is indicated by the presence of the genes necessary for nitrogen fixation (*nifKDH*) and the biosynthesis of thiamine, riboflavin, cobalamin, and a flagellum. Conversely, several traits confirmed during culture experiments were not observed in the genome, including genes for the reduction of Fe(III), such as *mtrABC*, and genes essential for carbon fixation (8).

### Accession number(s).

This whole-genome shotgun project has been deposited at DDBJ/ENA/GenBank under the accession number PPFX00000000. The version described in this paper is version PPFX01000000.
